# Effect of intramammary infusion of a carvacrol-based botanical product at dry-off on udder health in organic-certified dairy cows

**DOI:** 10.3168/jdsc.2026-1039

**Published:** 2026-05-22

**Authors:** P. Muñoz-Boettcher, C. Hernandez-Gotelli, C. Ibarguren, R. Weng Zeng, J. Velez, A. De Vries, E. Miller-Cushon, B.J. Heins, R.A. Lynch, Q. Kolar, G.M. Schuenemann, V. Cabrera, E. Silva, P. Pinedo

**Affiliations:** 1 Department of Animal Sciences, Colorado State University, Fort Collins, CO 80523; 2 Aurora Organic Farms, Platteville, CO 80651; 3 Department of Animal Sciences, University of Florida, Gainesville, FL 32611; 4 West Central Research and Outreach Center, University of Minnesota, Morris, MN 56267; 5 Department of Animal Sciences, Cornell University, Ithaca, NY 14850; 6 Department of Veterinary Preventive Medicine, The Ohio State University, Columbus, OH 43210; 7 Department of Animal and Dairy Sciences, University of Wisconsin–Madison, Madison, WI 53706; 8 Department of Plant Pathology, University of Wisconsin–Madison, Madison, WI 53706

## Abstract

The aim of this study was to evaluate the effect of intramammary infusion of a carvacrol-based botanical product at dry-off on udder health during the subsequent lactation in Holstein cows managed in an organic-certified system. This randomized negative-controlled trial included 159 Holstein cows calving from May to December 2024 at a commercial organic dairy. Before the final milking before dry-off, composite milk samples were collected for SCC and bacteriological culture diagnosis. Immediately after milking, a carvacrol-based botanical product was intramammarily infused into each quarter in the treatment group, whereas distilled water was infused to the control group. Following calving, composite milk samples were collected during periods 1 (2–14 DIM) and 2 (15–30 DIM). Multivariable mixed linear regression models for repeated measures were used to assess the effect of treatment on log_10_-transformed SCC (log_10_SCC) during periods 1 and 2. Multivariable mixed logistic regression models for repeated measures were fitted to evaluate the effect of treatment on the probabilities of (1) bacteriological cure (negative culture) at period 1 in cows infected at dry-off and (2) new IMI at period 1 in cows not infected at dry-off, by treatment group. At dry-off, 47.2% of cows were uninfected, whereas 16.4% and 22.6% were infected with *Staphylococcus aureus* and *Staphylococcus chromogenes*, respectively. After calving, these percentages were 56.9%, 18.5%, and 13.9%, respectively. No differences in log_10_SCC were observed between treatment groups during periods 1 and 2. Nevertheless, when comparing cows infected with *S. aureus* at dry-off, cows treated with the carvacrol-based botanical product showed higher log_10_SCC values than those in the control group in period 2. Conversely, the probability of bacteriological cure in cows infected at dry-off with the 3 pathogen groups (*S. aureus*, *S. chromogenes*, other pathogens), as well as the probability of new IMI at period 1 in cows not infected at dry-off, were similar between the treatment and control groups. The results indicated that the carvacrol-based botanical product was not effective in reducing either the log_10_SCC, the occurrence of new IMI, or the recovery of cows infected at dry-off. In conclusion, the intramammary infusion of a carvacrol-based botanical product at dry-off did not improve udder health in the subsequent lactation of dairy cows in an organic-certified system.

## Highlights

• *Staph. aureus and Staph. chromogenes* were the most common isolates in early lactation.
• The carvacrol-based product did not improve log_10_SCC in cows infected with *Staph. aureus* or *Staph. chromogenes*.
• Treatment did not reduce the likelihood of infection in early lactation.

## Graphical Abstract

**Figure:**
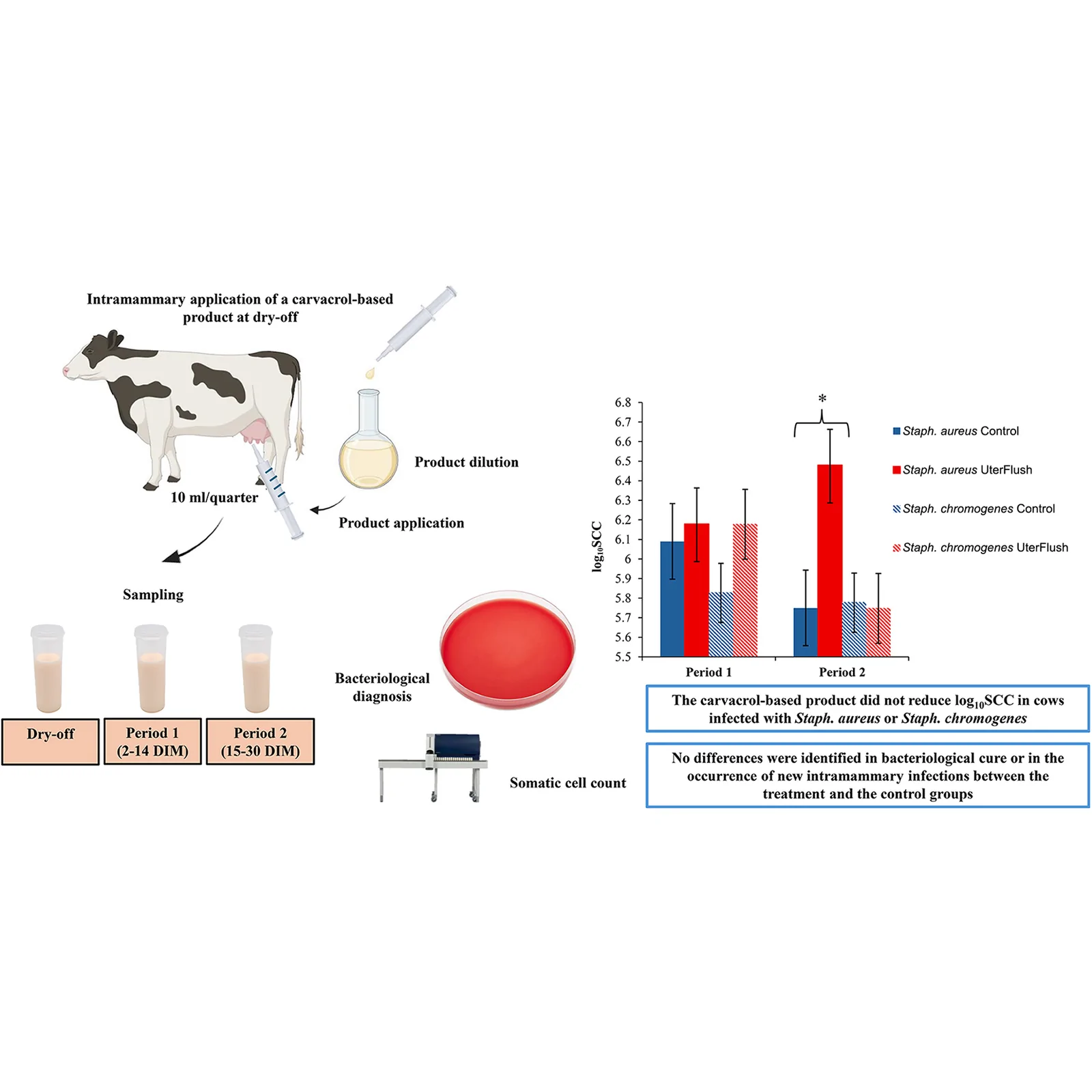
**Summary:** This study evaluated the effect of a carvacrol-based intramammary product administered at dry-off on udder health during the subsequent lactation in organically managed Holstein cows. This randomized controlled trial included 159 cows from 1 commercial organic dairy, which received either an intramammary infusion of a carvacrol-based product or distilled water at dry-off. Log_10_-transformed SCC (log_10_SCC) and bacteriological status were assessed at dry-off and during the first month of lactation. No differences were observed between treatment groups in log10SCC during periods 1 (2-14 days in milk [DIM]) and 2 (15-30 DIM), or in the probabilities of bacteriological cure and new intramammary infections. However, cows infected with *Staphylococcus aureus* and treated with a carvacrol-based product showed higher log_10_SCC in period 2. Overall, the carvacrol-based product did not improve udder health during dry-off in an organic system.

The organic dairy industry has grown steadily over the past 3 decades, largely in response to rising consumer demand for production systems that prioritize sustainability, biodiversity, and reliance on natural ecological processes to support healthy and productive agroecosystems (Müller et al., 2017; USDA-ERS, 2024). However, organic dairy farmers in the United States face challenges related to the use of therapeutic products, with substantial restrictions on available options for managing bacterial diseases (Ruegg, 2009) because the use of antimicrobial treatments results in permanent loss of the organic status of the treated animal.

Mastitis is a multifactorial disease that mainly results from IMI caused by bacteria (Barkema et al., 1999, 2006). This disorder remains the most common and costliest disease affecting dairy cows worldwide, compromising both cow welfare and survival in the system (Halasa et al., 2007). The relevance of mastitis to the dairy industry is underscored by the fact that the largest proportion of antimicrobials administered on conventional dairy farms is delivered via intramammary infusion for the treatment or prevention of mastitis (Pol and Ruegg, 2007). Nonetheless, the strict prohibition on the use of antimicrobials in US organic systems reduces the availability of therapeutic options for treating mastitis (Pol and Ruegg, 2007; USDA, 2021).

Organic dairy producers interviewed by Brock et al. (2021) indicated that their initial treatment for clinical mastitis was to strip the affected quarters, which promotes the removal of bacteria and bacterial toxins from the udder to improve healing. This management is usually combined with topical treatments containing peppermint (Brock et al., 2021), whose primary active compound, menthol, has been described as having anti-inflammatory properties (Liu et al., 2021). A survey of 20 organic farmers in Wisconsin reported the use of garlic tincture, aloe vera, or whey-based products as nontraditional antimicrobial alternatives (Pol and Ruegg, 2007; Ruegg, 2009). Finally, if mastitis persists after treatment, dairy producers typically dry-off the affected quarter or cull the cow (Brock et al., 2021).

The dry-off procedure provides an opportunity for both prophylactic management to prevent new infections and therapeutic interventions to clear existing infections. Some organic dairy farmers address this need by administering a variety of nonantimicrobial organic products (Pol and Ruegg, 2007; Poizat et al., 2017), but peer-reviewed studies that demonstrate their clinical efficacy are scarce (Ruegg, 2009). However, some organic treatments have shown promising results for treating infectious diseases affecting other organ systems. Pinedo et al. (2015) found that cows treated with an intrauterine infusion of Optimum UterFlush (Van Beek Natural Science, Orange City, IA) had a higher likelihood of recovering from toxic puerperal metritis and exhibited improved reproductive performance. UterFlush is an organic product containing carvacrol (4-isopropyl-2-methylphenol) derived from aromatic plants like oregano and used during the postpartum period to help maintain and restore a healthy uterine environment. Moreover, Sannmann et al. (2013) reported that the most frequent pathogens recovered from cows with toxic puerperal metritis were *Escherichia coli*, *Trueperella pyogenes*, and *Fusobacterium necrophorum*. Nevertheless, other microorganisms, such as *Pseudomonas* spp., *Streptococcus* spp., and *Staphylococcus* spp., may also affect the uterine environment, causing disease (Williams et al., 2005; Azawi, 2008; Santos et al., 2011). Thus, considering the similarities in bacterial species affecting both the uterine and mammary gland environments, it seemed plausible that an organic product used to treat a similar microbiological environment could positively affect the recovery of the mammary gland during the dry-off period. We hypothesized that intramammary infusion of a carvacrol-based botanical product at dry-off would improve udder health in the subsequent lactation, as reflected by lower SCC, a higher probability of bacteriological cure in cows infected at dry-off, and a lower occurrence of new IMI during early lactation, compared with untreated control cows in an organic dairy system. Therefore, the objective of this study was to evaluate the effect of intramammary infusion of a carvacrol-based botanical product, formulated to maintain and restore the normal uterine environment, on udder health in the subsequent lactation of Holstein cows managed in an organic-certified system.

This randomized negative-controlled trial included multiparous Holstein cows from a commercial, organic-certified dairy herd in northeast Colorado in the United States and was conducted from May to December 2024. On average, this farm has 10,800 lactating cows, divided into 4 units managed under a year-round calving system. A total of 201 cows were included in the study. Mean ± SD parity was 3.2 ± 1.3. The geometric mean SCC of the cows sampled at dry-off was 615,000 cells/mL (95% CI: 477,000–793,000). The arithmetic mean of the log_10_-transformed SCC (**log_10_SCC**) was 5.79 (95% CI: 5.68–5.90). Only cows without clinical mastitis and with all 4 functional quarters at the time of dry-off were included.

Cows were housed in freestall barns with sand-bedded stalls and had free access to dry-lot barns located outside the main facility. To comply with USDA organic dairy regulations, cows must spend at least 120 d grazing, with the distribution of these days varying depending on weather conditions, especially on warm and rainy days. Feed bunks were checked throughout the day, and refusals were monitored to make day-to-day adjustments based on DMI. The bedding was turned and scraped daily, manure removal from the barn's alleys was performed twice daily during the morning and night milkings, and scraping of the dry-lots was completed every other day. Bedding was fully cleaned and replaced once a year.

The dairy farm included in this study has a dry-off eligibility criterion for cows that includes days pregnant, DIM, and milk yield (<10 kg/d). Cows that met all these conditions were kept in a separate pen for 7 to 10 d, where they were milked once daily and received a reduced amount of DM. During the study, cows were enrolled in weekly batches and randomly assigned to the experimental groups upon entry into the rotary milking parlor. For example, 1 cow was placed in the treatment group, the next one in the control group, and so on. Before the last milking of the lactation, composite milk samples were aseptically collected by the research team, following the National Mastitis Council guidelines (Adkins et al., 2017) for SCC determination and bacteriological analysis. When cows scheduled for drying off were in the rotary milking parlor, the 4 functional quarters were forestripped, discarding at least 4 strips of milk per quarter. Subsequently, all teats were scrubbed and cleaned using paper towels soaked in 90% alcohol, using one towel per quarter. Then, 2 aseptic composite milk samples were collected: the first for bacteriological culture and the second for SCC determination. For each sample, 2 to 3 milk strips per quarter were collected into sterile sample tubes until each one was filled.

After milking, 10 mL of a carvacrol-based botanical preparation formulated for intrauterine administration was infused intramammarily into each quarter (treatment group; UterFlush, **UtF**), using the product dilution specified on the label (30 mL syringe mixed with 1,000 mL of distilled water). Therefore, 1 syringe was sufficient to treat 25 cows. Additionally, a control group (**CON**) was created for comparison, instilling 10 mL of distilled water in each quarter. Briefly, the procedure was performed as follows: First, each teat end was scrubbed and cleaned with alcohol pads (a separate pad for each teat); second, the application tube tip was inserted into the teat canal, and the administration doses (10 mL) were instilled into each quarter; then, the teats were massaged after treatment, and finally, all teats were covered with an iodine-based teat dipping solution following treatment. This procedure resembles the dry-off antibiotic administration routine on conventional dairy farms; however, in accordance with organic regulations, no internal or external teat sealants were used. After the product was administered, all the cows included in the study resumed their normal routine following the dry-off period (∼60 d).

Once cows began calving, they were sampled twice after milking. To do this, cows were handled in a chute located at the milking parlor exit. The first milk sample was collected within the first 14 d of lactation (period 1), and the second within d 15–30 of lactation (period 2). Subsequently, milk samples were transferred on the same day to a certified milk quality laboratory (The Dairy Authority, Greeley, CO). To assess SCC, collected milk samples were processed using a Fossomatic analyzer (FOSS, Hillerød, Denmark), which determines the SCC based on flow cytometry principles. For bacteriological analysis, 10 µL of milk was plated on one quadrant of a tryptic soy agar plate supplemented with 5% sheep blood and 0.1% esculin, and 100 µL of milk was streaked on one-half of a MacConkey agar plate, and both plates were incubated at 37 **°**C for 48 h. The bacterial genus was identified by colony morphology and biochemical testing, and the bacterial species were finally determined using MALDI-TOF MS. Spectra were compared against the manufacturer's reference database (Bruker Biotyper), and identification scores were interpreted according to standard thresholds (≥2.0 species-level identification and 1.7–1.99 for genus-level identification), as previously described in dairy microbiology studies (Nonnemann et al., 2019). Once microorganism species were confirmed, cows were classified as infected if any bacterial species were detected; as mixed infections when 2 different pathogens were identified in 1 composite milk sample; and as noninfected if milk cultures showed no bacterial growth. Last, milk samples were classified as contaminated when 3 or more different pathogen species were identified.

Cow demographic and health information, including parity number and calving and disease dates, were extracted from DART herd management software (Dairy Records Management Systems, Raleigh, NC). Thereafter, all the information was transferred into Microsoft Excel spreadsheets (Microsoft Corp., Redmond, WA) for further analysis. The sample size was calculated assuming a 13-percentage point difference in the prevalence of subclinical mastitis during the first month of lactation between groups. The expected prevalence in organic dairy herds was set at 23% based on values reported by Mullen et al. (2013) for organic dairies in the United States. Sample size calculations were performed using WINPEPI (version 11.62; Abramson, 2011), assuming a statistical power of 80%, which resulted in a minimum required sample size of 99 cows per group.

Statistical analyses were performed in R, version 4.2.2 (R Core Team, 2022), using the nlme (Pinheiro et al., 2024), lme4 (Bates et al., 2015), and emmeans (Lenth, 2022) packages. Initial data exploration and descriptive analyses were performed through visual assessment of the variables included in the study. Because SCC were not normally distributed, they were log_10_-transformed to address this problem. To evaluate the effect of treatment on log_10_SCC dynamics, data were analyzed for the 2 follow-up periods (period 1 and period 2). Conversely, to assess the probability of bacteriological cure (negative culture) in cows infected at dry-off and new IMI in cows not infected at dry-off, data were analyzed only in period 1. Multivariable mixed linear regression models for repeated measures were used to calculate the estimated marginal means for log_10_SCC (outcome variable), with treatment as the main predictor and its interaction with assessment period, adjusting for log_10_SCC at dry-off and days dry as covariates. Cow identification (**ID**) was included as a random effect to account for repeated measures. To account for the nonindependence of repeated measurements within individual cows, a compound symmetry correlation structure (corCompSymm) was included in the mixed-effects models using the lme function from the nlme package in R.

Multivariable mixed logistic regression models for repeated measures were fitted to assess potential associations of 2 outcome variables: (1) the probability of bacteriological cure (negative culture) in period 1 by treatment group among cows infected at dry-off, adjusting for pathogen group at dry-off and days dry as covariates, and (2) the probability of new IMI at period 1 in cows not infected at dry-off, by treatment group, adjusting for days dry as covariate. Cow ID was included as a random effect in both models to account for repeated measures.

Because the prevalence of IMI in milk samples was mainly attributed to the presence of both *S. aureus* and *S. chromogenes* (Table 1), and considering that these pathogens are among the most important staphylococcal species causing IMI, species-specific analyses were performed to evaluate the previously described outcome variables. Based on the distribution of pathogens, 3 groups were defined: *S. aureus*, *S. chromogenes*, and other pathogens, which included all less prevalent species identified in the samples.

**Table 1:** Distribution of pathogens in 456 composite milk samples obtained from 159 cows at dry-off, and during period 1 (2–14 DIM) and period 2 (15–30 DIM)

Pathogen	Sampling time			Total	Total (%)
	Dry-off	Period 1	Period 2		
*Acinetobacter pseudolwoffii*	—	—	1	1	0.2
*Corynebacterium amycolatum*	2	—	—	2	0.4
*Corynebacterium casei*	—	1	—	1	0.2
*Corynebacterium* spp.	—	1	2	3	0.7
*Enterococcus faecium*	—	1	—	1	0.2
*Escherichia coli*	2	1	—	3	0.7
*Staphylococcus aureus*	26	28	37	91	20
*Staphylococcus auricularis*	1	—	—	1	0.2
*Staphylococcus chromogenes*	36	21	18	75	16.4
*Staphylococcus epidermidis*	1	—	2	3	0.7
*Staphylococcus haemolyticus*	1	—	—	1	0.2
*Staphylococcus hyicus*	—	1	—	1	0.2
*Staphylococcus simulans*	1	1	—	2	0.4
*Staphylococcus xylosus*	2	1	—	3	0.7
*Streptococcus dysgalactiae*	—	1	2	3	0.7
*Streptococcus* spp.	—	—	1	1	0.2
*Streptococcus uberis*	—	3	1	4	0.9
*Trueperella pyogenes*	1	—	1	2	0.4
Contaminated	2	2	3	7	1.5
Mixed infections	9	3	1	13	2.9
No growth	75	86	77	238	52.2
Total (n)	159	151	146	456	—
Total (%)	34.9	33.1	32.0	100	100

Log_10_-transformed SCC and probabilities of bacteriological cure and new IMI between treatment groups were compared using Tukey-adjusted pairwise comparisons for each period. Results are reported in estimated marginal means and probabilities. Assumptions of constant variance and normal residuals were assessed by residual diagnostic plots. Statistical significance was established at *P* ≤ 0.05 level using a likelihood ratio test.

Initially, 201 cows were enrolled in the experiment. However, 42 cows (18 UtF and 24 CON) were removed from the analysis based on exclusion criteria that included culling during the dry period. During periods 1 and 2, the total number of cows decreased because not all animals could be sampled due to logistical constraints. For instance, some cows were relocated to other pens (n = 8), whereas others were culled shortly after calving due to disease (n = 5). Consequently, it was not possible to maintain the same number of samples obtained at the dry-off throughout the study. After data cleaning, the analysis included information from 159 Holstein cows sampled from May to December 2024. A total of 456 composite milk samples were included in the analysis. As shown in Table 1, the most frequently isolated gram-positive pathogens were *S. aureus* (20%), *S. chromogenes* (16.4%), *Streptococcus uberis* (0.9%), and *S. epidermidis* (0.7%). The most prevalent gram-negative bacteria included *Escherichia coli* (0.7%) and *Acinetobacter pseudolwoffii* (0.2%). No bacterial growth was detected in 52.2% of the samples. Furthermore, mixed infections and contaminated diagnoses were identified in 2.9% and 1.5% of the samples, respectively.

The total percentage of cows with IMI at dry-off was 56% and 46% in the CON and UtF groups, respectively. The cure rate for *S. aureus* infections was 16% in the CON group and 10% in the UtF group. For *S. chromogenes*, the cure rates were 35% and 43% in the CON and UtF groups, respectively (Table 2). New IMI rates in the CON group were 3.4% for *S. aureus* and 1.1% for *S. chromogenes*, whereas in the UtF group they were 8.8% and 4.8%, respectively. No differences in new IMI rates were established between the 2 treatment groups.

**Table 2:** Model-estimated probabilities (±SE) for 2 outcomes: (1) bacteriological cure (negative culture) at period 1 (2–14 DIM) in cows infected at dry-off, and (2) new IMI at period 1 in cows not infected at dry-off, by treatment group (CON = control group; UtF = UterFlush group)

Dry-off infection status	Treatment group				*P*-value
	CON		UtF		
	Probability	SE	Probability	SE	
Infected at dry-off and cured at period 1^1^					
*S. aureus*	0.16	0.11	0.10	0.10	0.67
*S. chromogenes*	0.35	0.12	0.43	0.13	0.65
Other pathogens	0.78	0.14	0.77	0.14	0.98
Not infected at dry-off and infected at period 1^2^					
Total pathogens	0.26	0.08	0.20	0.06	0.53

1 Model included the interaction between treatment and pathogen group at dry-off as the main predictors, days dry as a fixed effect, and cow ID as a random effect.

2 Model included treatment as the main predictor, days dry as a fixed effect, and cow ID as a random effect.

The epidemiology of IMI on organic dairies has not been widely studied. However, the distribution of pathogens isolated from composite milk samples during the study period was similar to that reported in previous studies. As presented by Pol and Ruegg (2007), the prevalence of most mastitis pathogens at the quarter level was higher in organic (51.2%) than in conventional farms (31.5%) in the United States. In addition, quarters from organic farms were more likely to be positive for *S. aureus* (Pol and Ruegg, 2007). Indeed, this microorganism can be considered as one of the most frequently isolated *Staphylococcus* species in organic dairy herds (Peña-Mosca et al., 2023; Jeffrey et al., 2025). Likewise, our results indicate that this pathogen accounted for 20% of samples collected during the assessment period, corresponding to the most prevalent IMI. This higher prevalence may be associated with restrictions on organic farms regarding the use of antimicrobials (NMC, 2019). Conversely, organic dairies have been described to have a higher average parity, which is associated with an increased prevalence of contagious mastitis pathogens such as *S. aureus* (De Vliegher et al., 2012). Furthermore, *S. chromogenes* was identified as the second-most prevalent pathogen species, accounting for 16.4% of samples collected during the assessment period. In a convenience sampling study that included 5 organic farms in the United States, Peña-Mosca et al. (2023) reported that *S. chromogenes* was the most isolated NAS species (average 22.8%) during the first 5 wk of lactation, with a higher prevalence in first-lactation cows than in multiparous cows. Similarly, in a convenience sample of 10 herds in Vermont, in the United States, Jeffrey et al. (2025) described *S. chromogenes* as the most common staphylococcal species, accounting for 59% of the IMI in organic cows.

Overall, the UtF group showed higher log_10_SCC than the CON group (Figure 1), but these differences were not significant at any assessment period (*P* = 0.32). Pathogen-specific analyses revealed no differences between treatment groups in cows infected with *S. aureus* during period 1. In contrast, UtF cows infected with *S. aureus* exhibited higher log_10_SCC than CON cows during period 2 (*P* = 0.01). No differences between treatment groups were observed in cows infected with *S. chromogenes* across assessment periods. These findings contrast with previous studies reporting that carvacrol-based botanical products possess anti-inflammatory, antioxidant, and antibacterial properties, which could potentially reduce SCC (Friedman, 2014; Suntres et al., 2015). This discrepancy may be related to differences in pathogen dynamics, treatment timing, or study conditions. Therefore, the applicability of these results to other herds may depend on factors such as pathogen prevalence, management practices, and environmental conditions.

**Figure 1:**
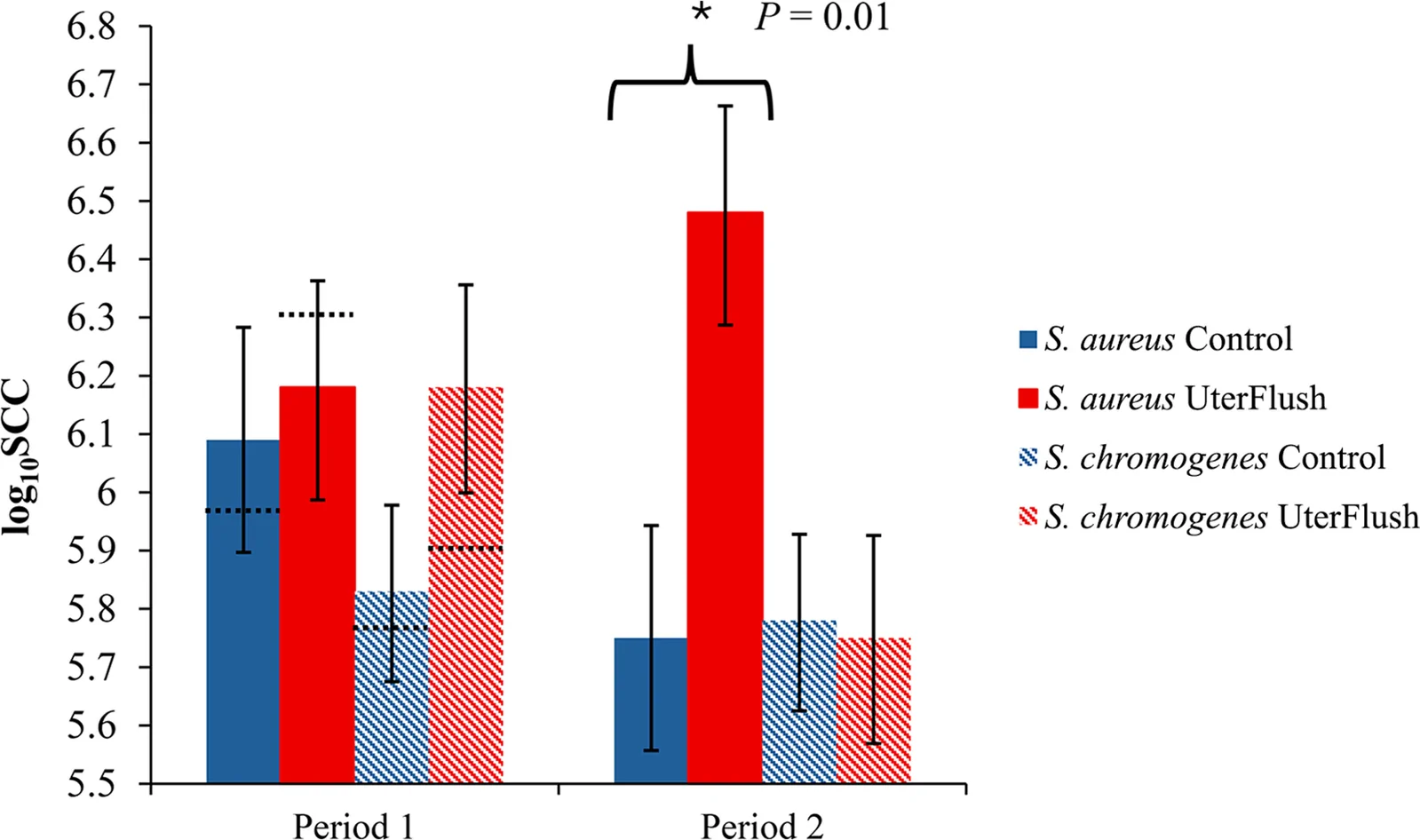
Estimated marginal means (± SE) of log_10_SCC at periods 1 (2–14 DIM) and 2 (15–30 DIM) in cows diagnosed with *S. aureus* (CON: blue bar; UtF: red bar) and *S. chromogenes* (CON: blue dashed bar; UtF: red dashed bar) at dry-off. The model included interaction between treatment and period as the main predictors, logSCC at dry-off, and days dry as fixed effects, and cow ID as a random effect. Cows infected by *S. aureus* started with a dry-off log_10_SCC mean (dotted lines) of 5.97 and 6.31 for the CON and UtF groups, respectively. Cows infected by *S. chromogenes* started with a dry-off log_10_SCC mean (dotted lines) of 5.77 and 5.88 for the CON and UtF groups, respectively. Asterisk within a period indicates significant differences (*P* < 0.05). The number of observations (n) contributing to each mean was as follows: *S. aureus*, CON (period 1 = 13; period 2 = 13) and UtF (period 1= 12; period 2 = 11); for *S. chromogenes*, CON (period 1 = 17; period 2 = 15) and UtF (period 1 = 16; period 2 = 15).

When analyzing the probability of a negative bacteriological culture during period 1 in cows infected at dry-off, similar probabilities were observed between the UtF and CON groups, regardless of pathogen group (Table 2). Similarly, when analyzing cows not infected at dry-off, no differences in the probability of new IMI during period 1 were observed between treatment groups. Peña-Mosca et al. (2023) reported high persistence in IMI caused by *S. aureus* (59.7%) and *S. chromogenes* (68.8%) during lactation. Although it has been noted that most NAS species have poor adaptation to the mammary gland and are mostly environmental in their epidemiology (De Buck et al., 2021), *S. chromogenes* could be better adapted to survive in the udder than other NAS species. Indeed, half of the *S. chromogenes* IMI persisted compared with only 16.4% of IMI caused by the other NAS species (Valckenier et al., 2021).

The bacteria most commonly isolated from IMI include *S. aureus*, *Streptococcus* spp., NAS, and coliform species (Pol and Ruegg, 2007). Conversely, Santos et al. (2011) reported that microorganisms, such as *Pseudomonas* spp., *Streptococcus* spp., and *Staphylococcus* spp., may affect the uterine environment, and that pathogens isolated from different anatomical sites in cows may share certain similarities. As previously described, treatments options at dry-off in organic dairy systems are limited. Products reported by farmers include ultrafiltered bovine whey, vitamin and microbial supplements, and aloe vera-based products; however, their use is subject to regulatory restrictions, and some are permitted only under specific conditions (Ruegg, 2009). Although we did not observe significant differences between the treatment groups, we anticipated potential benefits for udder health in cows treated with a carvacrol-based botanical treatment, based on a previous report showing a positive effect on uterine health (Pinedo et al., 2015). The main component of this plant-based product is carvacrol, a monoterpenic phenol synthesized by aromatic plants such as oregano. This compound has been reported to have antioxidative, anti-inflammatory, and antibacterial properties. Indeed, Pinedo et al. (2015) reported that cows affected by toxic puerperal metritis treated with carvacrol had higher odds of recovery and improved reproductive performance than control cows. Furthermore, it is important to note that the dilution used in our study was consistent with the manufacturer's recommendations for intrauterine use. Nevertheless, given that no previous studies have evaluated this product in relation to udder health, we opted for a lower and safer dose for the cows. However, future studies could explore higher concentrations to assess whether they might lead to improved udder health outcomes.

The current study provides new insights into a drying-off treatment option that could eventually have implications for both organic and conventional dairies aiming to reduce overall antimicrobial use on dairy farms because research on this topic remains scarce. Nonetheless, some limitations exist. First, the carvacrol-based botanical product evaluated in this study is not approved by the US Food and Drug Administration (**FDA**) for intramammary use, and its administration at dry-off constituted an off-label application conducted exclusively under controlled research conditions. All treatments were performed at dry-off, and cows remained in the dry period for approximately 60 d before calving. Therefore, no milk from treated cows entered the human food supply during the period immediately following treatment, and no animals were intended to be marketed for slaughter during the dry period. Pharmacokinetic studies showed that thymol and carvacrol exhibit rapid depletion following intramammary administration. Armorini et al. (2016) reported low but measurable residue levels at 96 h for liver and 36 h for milk, with short elimination half-lives (<3 h), and Mason et al. (2018) indicated half-lives in plasma of 1.7 h. Mason et al. (2017) evaluated the pharmacokinetics of carvacrol in mid-lactation dairy cows following intramammary infusion of Phyto-Mast and reported a plasma elimination half-life of 1.47  ±  0.79 h, with tissue elimination half-lives lower than 25.7 h. Thus, the duration of the dry period (∼60 d) substantially exceeded reported residue depletion intervals and functioned as a conservative withdrawal period before the subsequent lactation. Although these measures were implemented to mitigate food safety risks, US regulatory authorities may still consider residues of such compounds in edible tissues or milk as adulteration (Armorini et al., 2016). Because this study was designed solely to evaluate effectiveness and not to support regulatory approval or generate safety data for commercial use, an Investigational New Animal Drug (INAD) file through the FDA Center for Veterinary Medicine was not obtained. Therefore, these findings should not be interpreted as a recommendation for on-farm use, but rather as a contribution to the limited body of peer-reviewed evidence evaluating therapeutic alternatives for mastitis control in organic-certified systems under controlled research conditions. Future studies intended to support commercial or therapeutic claims should include formal pharmacokinetic evaluations and be conducted in consultation with the FDA. Second, during the dry-off procedure, we collected composite milk samples from the cows enrolled in the study. As reported by Reyher and Dohoo (2011), the use of composite milk samples may lead to an underestimation of the prevalence of IMI due to a dilution effect; therefore, some quarter IMI could be missed. Lastly, regular SCC were not available, preventing us from assessing the udder health of the enrolled cows and determining disease chronicity, which would help us perform a more accurate assessment.

The current findings do not support the use of a carvacrol-based botanical product administered by intramammary infusion at dry-off as an intervention for mastitis control under the conditions evaluated. Application of this product did not influence either the recovery or maintenance of mammary gland health during the subsequent lactation of dairy cows managed in an organic system.
